# Who’s at Risk? A Prognostic Model for Severity Prediction in Pediatric Acute Pancreatitis

**DOI:** 10.1097/MPG.0000000000002807

**Published:** 2020-10

**Authors:** Peter R. Farrell, Lindsey Hornung, Peter Farmer, Angelica W. DesPain, Esther Kim, Ryan Pearman, Beemnet Neway, Ashley Serrette, Sona Sehgal, James E. Heubi, Tom K. Lin, Jaimie D. Nathan, David S. Vitale, Maisam Abu-El-Haija

**Affiliations:** * Division of Gastroenterology, Hepatology and Nutrition, Cincinnati Children’s Hospital Medical Center, Cincinnati, OH; † Division of Biostatistics and Epidemiology, Cincinnati Children’s Hospital Medical Center, Cincinnati, OH; ‡ Pediatric Residency Program, Children’s Hospital of the King’s Daughters, Eastern Virginia Medical School, Norfolk, VA; § Department of Pediatrics, Eastern Virginia Medical School, Norfolk, VA; ∥ Division of Emergency Medicine, Children’s National Hospital, Washington, DC; ¶ Division of Critical Care Medicine, Children’s National Hospital, Washington, DC; # Division of Emergency Medicine, Cohen Children’s Medical Center, New Hyde Park, NY; ** Division of Gastroenterology, Hepatology and Nutrition, Children’s National Hospital, Washington, DC; †† Department of Pediatrics, University of Cincinnati College of Medicine, Cincinnati Children’s Hospital Medical Center, Cincinnati, OH; ‡‡ Division of Pediatric General and Thoracic Surgery, Cincinnati Children’s Hospital Medical Center, Cincinnati, OH; §§ Department of Surgery, University of Cincinnati College of Medicine, Cincinnati, OH.

**Keywords:** blood urea nitrogen, pediatrics, severe acute pancreatitis

## Abstract

**Objectives:**

The aim of the study was to validate and optimize a severity prediction model for acute pancreatitis (AP) and to examine blood urea nitrogen (BUN) level changes from admission as a severity predictor.

**Study Design:**

Patients from 2 hospitals were included for the validation model (Children’s Hospital of the King’s Daughters and Children’s National Hospital). Children’s Hospital of the King’s Daughters and Cincinnati Children’s Hospital Medical Center data were used for analysis of BUN at 24 to 48 hours.

**Results:**

The validation cohort included 73 patients; 22 (30%) with either severe or moderately severe AP, combined into the all severe AP (SAP) group. Patients with SAP had higher BUN (*P* = 0.002) and lower albumin (*P* = 0.005). Admission BUN was confirmed as a significant predictor (*P* = 0.005) of SAP (area under the receiver operating characteristic [AUROC] 0.73, 95% confidence interval [CI] 0.60–0.86). Combining BUN (*P* = 0.005) and albumin (*P* = 0.004) resulted in better prediction for SAP (AUROC 0.83, 95% CI 0.72–0.94). A total of 176 AP patients were analyzed at 24–48 hours; 39 (22%) met criteria for SAP. Patients who developed SAP had a significantly higher BUN (*P* < 0.001) after 24 hours. Elevated BUN levels within 24 to 48 hours were independently predictive of developing SAP (AUROC: 0.76, 95% CI: 0.66–0.85). Patients who developed SAP had a significantly smaller percentage decrease in BUN from admission to 24 to 48 hours (*P* = 0.002).

**Conclusion:**

We externally validated the prior model with admission BUN levels and further optimized it by incorporating albumin. We also found that persistent elevation of BUN is associated with development of SAP. Our model can be used to risk stratify patients with AP on admission and again at 24 to 48 hours.

A cute pancreatitis (AP) represents a significant disease burden in the pediatric population, the estimated incidence has been increasing over the past 2 decades and has now stabilized to greater than 1 in 10,000, with significant health and economic implications as well (1,2). Although there has been increased attention to pediatric AP in recent years, pediatric data regarding optimal management remain limited. The first management guidelines for pediatric AP were published by the North American Society for Pediatric Gastroenterology, Hepatology and Nutrition (NASPGHAN) in 2018 (3). This society effort noted several potential predictive models of severity of pediatric AP were previously published, some of which had varying specificity and sensitivity upon validation (3). The authors concluded further investigation was required to identify predictive markers of severity on admission with models that can be replicated at independent sites (3). In 2017, the NASPGHAN Pancreas Committee published severity classification guidelines for pediatric AP, providing a consensus definition of mild, moderately severe, and severe AP (SAP) in pediatrics for the first time (4). Now that a commonly shared definition of severity has been established, predictive models that use this classification are needed.

Following the publication of the 2017 severity guidelines, Vitale et al (5) developed a model to predict severity utilizing internal data from a prospective first attack-AP registry at Cincinnati Children’s Hospital Medical Center (CCHMC) (5). The model characterized patients based on the severity of their disease and examined clinical parameters on admission in an attempt to identify predictors of AP severity (5). From their model-building efforts, they identified blood urea nitrogen (BUN) on admission as a significant predictor of the development of severe disease in the pediatric population (5). BUN change after fluid resuscitation has been shown to predict severity in prior adult studies, but to our knowledge has not been studied in children (6,7).

The primary aim of this study was to validate and optimize the previously reported model using a separate cohort of patients from 2 distinct children’s hospitals, and the secondary aim was to further reexamine the role of BUN change after admission (5). Through resuscitation and fluid management, BUN shifts may occur, and the change in BUN has not been studied as a predictor of severity in pediatric AP. Our secondary aim was to evaluate whether BUN alone after 24 hours of resuscitation would be an independent predictor AP severity.

## METHODS

There were 3 distinct patient populations used to achieve the goals of the study.

*For the external validation and optimization cohort patients* were included from 2 sites: The Children’s Hospital of the King’s Daughters (CHKD) in Norfolk, VA, and Children’s National Hospital (CNH) in Washington, DC, obtained data from patients younger than 19 years from 2012 to 2018. Patients admitted to CHKD were identified retrospectively using billing codes from 2012 to 2017. Patients prospectively enrolled in an ongoing study of first time AP (Clincaltrials.gov: NCT03232473) at CNH from 2016 to 2018 were also included. This was designed to externally validate the model previously constructed at CCHMC, and thus data from CCHMC were not included.

For the BUN change analysis, we used collected data from CCHMC and CHKD cohorts. Data from CNH were not included because the patients were part of an ongoing randomized control trial and we did not wish to unintentionally prejudice the results of that study.

Permission was granted by each local institutional review board (IRB). The local measures for identifying patients at each institution are specified below.

The CHKD data represent a retrospective chart review of pediatric patients who presented to the emergency department or the inpatient ward diagnosed with AP. Permission was granted by the local IRB (CHKD IRB 17-04-WC-0096). Patients diagnosed with AP based on International Classification of Diseases, ninth revision (*ICD-9*) or *ICD-10* codes in either the emergency department or inpatient hospital ward were included. The following codes were used: *ICD-9* Codes = 577.0–577.2, 577.8–577.9; *ICD-10* Codes = K85.00–K85.02, K85.10–K85.12, K85.20–K85.22, K85.30–K85.32, K85.80–K85.82, and K85.90–K85.92.

The CNH data were obtained prospectively as part of an ongoing randomized controlled trial examining intravenous fluid choice in pediatric AP, which is registered at Clinicaltrials.gov (NCT03242473) between 2016 and 2018. Patients with first episode of AP were identified and enrolled in the study and the laboratory values on presentation, before randomization and the ultimate development of mild AP, moderate AP, or SAP were included in the analysis. Permission was granted by the local IRB for the study (CNH IRB Pro00007698). As previously described, the data from CCHMC was prospectively collected on patients who presented with their first episode of AP between March 2013 and January 2017 (CCHMC IRB 2012–4050) (5).

For all of the datasets, the first laboratory values for each patient obtained within 24 hours of presentation were included in the analysis. In all patients, the diagnosis of AP was confirmed using the International Study Group of Pediatric Pancreatitis: In Search for a Cure (INSPPIRE) group diagnostic criteria for pediatric AP originally published in 2012, which have subsequently been endorsed by the Pancreas Committee of NASPGHAN (3,8). Diagnosis required a patient to meet at least 2 of the following 3 criteria: characteristic abdominal pain, amylase or lipase ≥3 times the upper limit of normal for age, and imaging findings consistent with AP (eg, edema, necrosis, hemorrhage, abscess, pseudocyst) (3,8). Severity of AP was classified utilizing the 2017 NASPGHAN criteria, based on the presence or absence of local pancreatic complications or transient (<48 hours) versus persistent (≥48 hours) systemic organ dysfunction (4). Mild AP was defined as the absence of local pancreatic or systemic organ dysfunction and the absence of exacerbation of underlying disease (4). Moderately severe AP was defined as transient (<48 hours) organ dysfunction, local pancreatic complications, or exacerbation of underlying disease (4). SAP is defined as involving prolonged (≥48 hours) organ dysfunction (4). Moderately severe AP and SAP were included in a single group labeled SAP for the purposes of statistical analysis to separate the mild cases from the nonmild cases.

### Statistical Analysis Methods

Data were analyzed using SAS, version 9.4 (SAS Institute, Cary, NC). Because of skewed distributions, continuous data were summarized as medians with interquartile ranges (IQR: 25th–75th percentiles), whereas categorical data were summarized as frequency counts and percentages. If laboratory values were reported as below the limit of detection, the values used for analysis were the limit of detection value divided by the square root of 2 (9). For continuous data, nonparametric Wilcoxon-Mann-Whitney tests were used to compare characteristics and laboratory values between groups. Chi-square and Fisher exact tests were used, as appropriate, for group comparisons of categorical data. Logistic regression models were used for the validation model and in the model-building process. Stepwise selection was performed to identify variables to optimize prediction of developing SAP when combined in a multivariable logistic regression model based on significant *P* values and the receiver operating characteristics (ROC) curve. A *P* value <0.05 was considered statistically significant.

## RESULTS

### Validation of the Severity Model

During the study period, a total of 57 patients at CHKD met the criteria for first episode of AP. There were a total of 16 patients recruited from CNH who met the diagnostic criteria for first episode of AP. For the validation of the previously described model, these 73 total patients were included for analysis (51 mild AP, 15 moderately severe AP, and 7 SAP cases). Moderately severe AP and SAP were included in a single group labeled SAP for this analysis. From CHKD, 16 of 57 patients were included in the SAP cohort, and from CNH, 6 of 16 were included in the SAP cohort.

#### Baseline Clinical and Biochemical Laboratory Values on Admission Between Mild Acute Pancreatitis and Severe Acute Pancreatitis Cases

Table 1 shows baseline characteristics of the patients in this cohort segregated by AP severity. Patients with mild AP had similar baseline characteristics compared to SAP group. The SAP group had significantly longer length of stay (LOS), with a median LOS of 147.5 hours (IQR 70–348 hours), compared to the mild AP group who had a median LOS of 94 hours (IQR 63–154 hours) (*P* = 0.0497, Table 1). Table 1 also includes a selected sample of laboratory values obtained on admission and the results of the univariate analyses performed to test for group differences. BUN and serum albumin significantly differed between the SAP and mild AP groups (Table 1). One hundred percent of the patients in the dataset had a BUN level measured at the time of admission, and there was a significant difference between BUN levels in the SAP (median 14.5 mg/dL, IQR 11–19 mg/dL) and mild AP (median 11 mg/dL, IQR 813 mg/dL) groups (*P* = 0.002, Table 1). More than 90% of the patients had a serum albumin level measured on admission, and the values were significantly higher in the mild AP (median 4.0 g/dL, IQR 3.6–4.6 g/dL) group when compared to the SAP (median 3.3 g/dL, IQR 3.1–4.2 g/dL) group (*P* = 0.005, Table 1). The complete list of biochemical parameters examined on admission is included as Supplemental Table 1 (Supplemental Digital Content, http://links.lww.com/MPG/B859).

#### Higher Values for Blood Urea Nitrogen and Lower Values for Albumin Represent Increasing Levels of Severity

**FIGURE 1.**
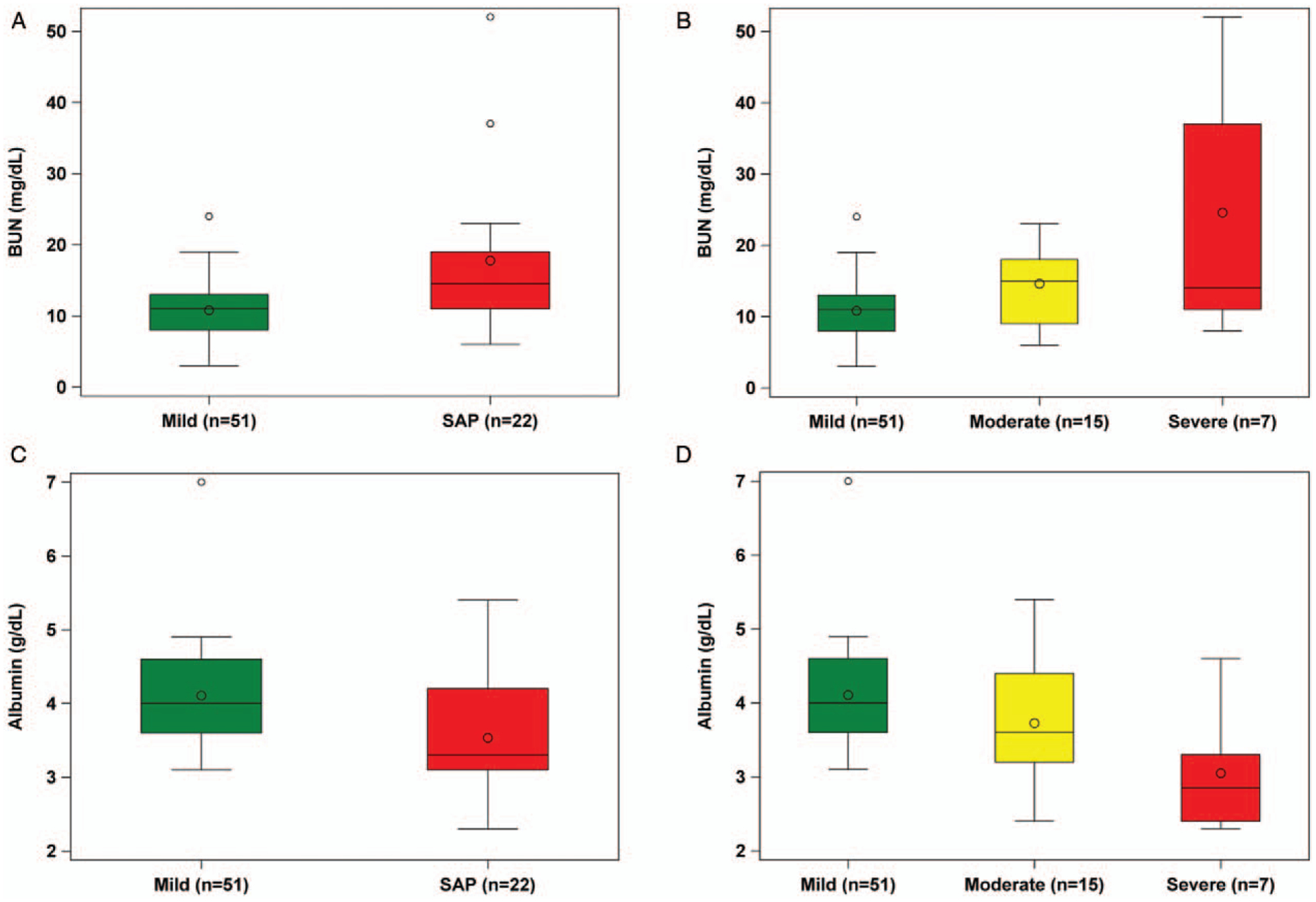
Boxplots examining the relationship of variables identified from the validation cohort from Children’s Hospital of the King’s Daughters (CHKD)/Children’s National Hospital (CNH) (SAP: Combined moderately severe and severe AP group). A, BUN levels on admission in the mild AP and SAP group. B, Admission BUN levels separated based on all forms of severity (mild, moderately severe, and severe). C, Albumin levels on admission in the mild AP and SAP group. D, Admission albumin levels separated based on all forms of severity (mild, moderately severe, and severe). BUN = blood urea nitrogen; SAP = severe acute pancreatitis.

We explored the relationship of BUN and albumin levels across all of the severity classifications (mild, moderately severe AP and SAP), and found a trend toward higher BUN levels and lower albumin levels from mild to moderately severe to the most severe AP group. We found that as the BUN value increased, so did the likelihood of developing SAP (Fig. 1 A and B). In addition, as serum albumin decreased, the likelihood of developing SAP increased (Fig. 1C and D).

#### Validation of the Blood Urea Nitrogen Model

**FIGURE 2.**
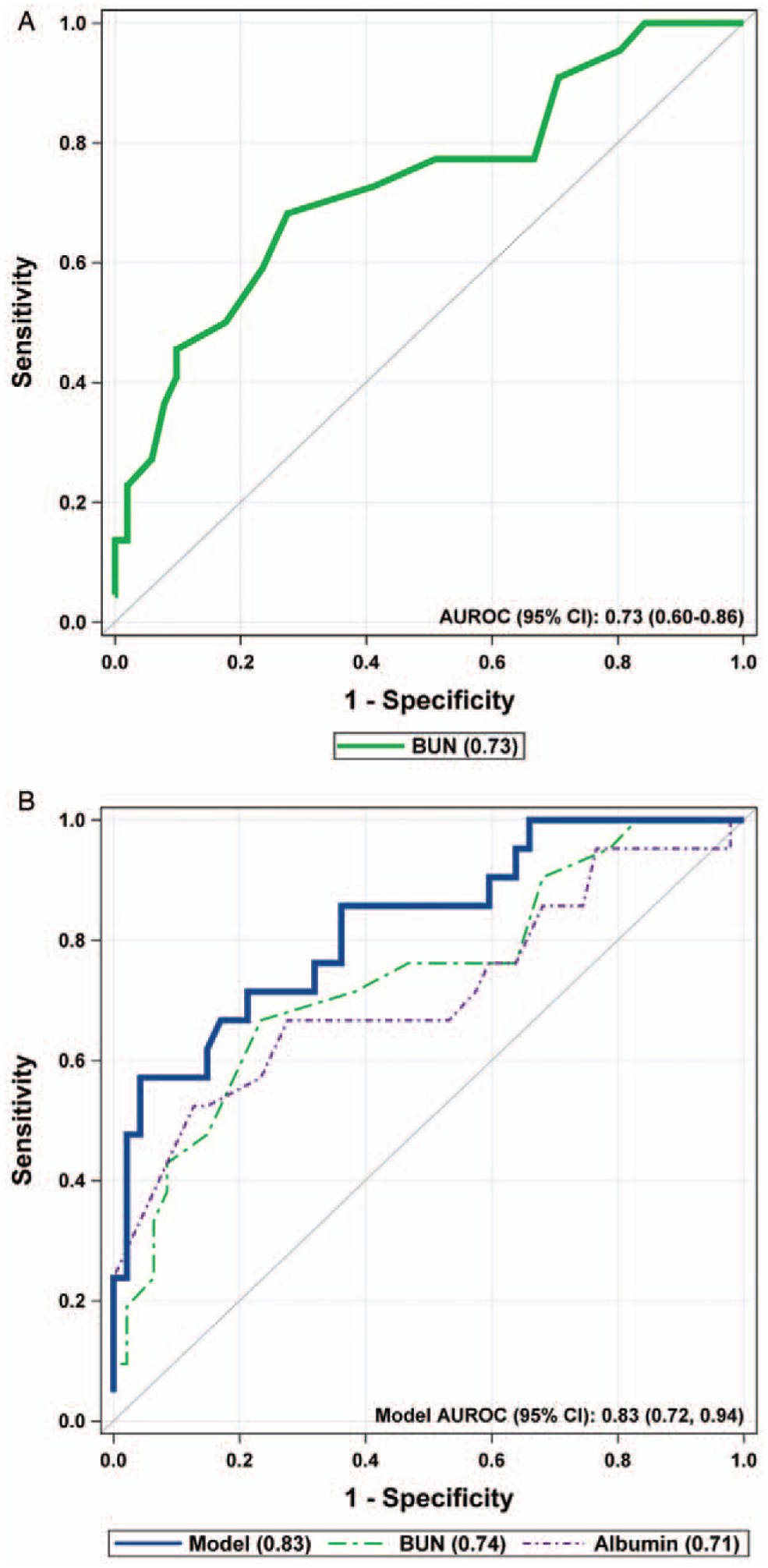
Results of the external validation cohort. A, AUROC of the multivariable model from validation cohort, BUN on admission is a significant predictor of SAP with an AUROC of 0.73. B, Model optimization with BUN + albumin on admission, AUROC 0.83. When combined in a multivariable model, BUN (*P* = 0.005) and albumin (*P* = 0.004) are significant predictors of severe acute pancreatitis SAP (n = 73). AUROC = area under the receiver operating characteristic; BUN = blood urea nitrogen; CI = confidence interval.

The previously reported model had identified BUN as a significant prognostic marker of severity (area under the receiver operating characteristic [AUROC] curve: 0.75, 95% confidence interval [CI] 0.61–0.89); therefore, one of our aims was to validate BUN as a predictor of severity using a separate cohort of patients (5). We were able to validate the previous model, as BUN was found to be a significant predictor of any form of SAP in our cohort (Fig. 2A) (AUROC curve: 0.73, 95% CI 0.60–0.86, *P* = 0.005, sensitivity 68%, specificity 73%, positive predictive value [PPV]: 52%, negative predictive value [NPV]: 84%). These results mirrored the previously generated model values. The cutoff for development of SAP that optimized sensitivity and specificity in the model was a BUN of 13 mg/dL, with increasing specificity as the BUN level increased.

#### Optimization of the External Validation Model

To optimize the BUN model using stepwise selection and including other potential predictor variables from the external validation cohort into a multivariable logistic regression model, we found BUN (*P* = 0.005) and adding serum albumin (*P* = 0.004) created a better predictive model for SAP (AUROC curve: 0.83, 95% CI: 0.72–0.94, sensitivity 71%, specificity 79%, PPV 60%, NPV 86%) (Fig. 2B). Threshold admission values of BUN >13 mg/dL and serum albumin <3.6 g/dL were associated with an increased probability of developing SAP.

Akaike’s Information Criterion (AIC) was calculated for the models and evaluated to help determine which model would perform the best, with a lower score suggesting a better model. For the BUN only model, the AIC was 79.4. For the albumin only model, the AIC was 79.1. For the combined BUN and albumin model, the AIC was 66.2. Therefore, the BUN and albumin combined had the best AIC score, superior AUROC, and significant *P* values for both BUN and albumin, suggesting that BUN and albumin on admission optimized the model predictive capability.

#### Blood Urea Nitrogen Change at 24 to 48 Hours Exploration

**FIGURE 3.**
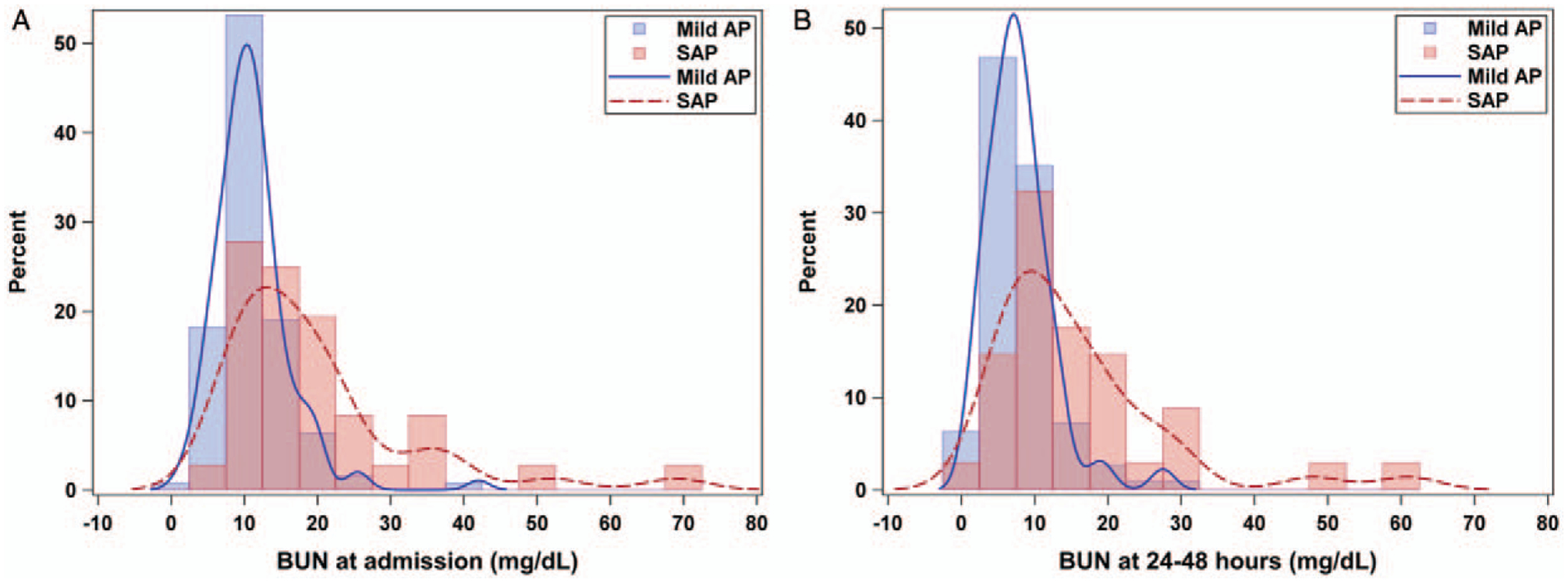
Kernel distribution of BUN levels at admission and at 24 to 48 hours. BUN levels are elevated in a higher percentage of SAP patients on admission (A), and this elevation is persistent (B) at 24 to 48 hours (n = 176). AP = acute pancreatitis; BUN = blood urea nitrogen; SAP = severe pancreatitis.

A total of 176 primary patients with AP were included for the purpose of this analysis from the 2 study sites as specified in the method section. Thirty-nine patients (22%) met criteria for SAP. For the clinical presentation and management; there was no statistical difference based on age, sex, rate or type of fluid used (isotonic, hypotonic, or total parenteral nutrition [TPN]) in the first 24 hours between the SAP and mild AP groups (Supplemental Table 2, Supplemental Digital Content, http://links.lww.com/MPG/B859). The SAP group had elevated BUN levels on admission (*P* < 0.001); SAP: median (IQR) of 15.5 (11.0–22.5) versus mild AP: 10.0 (8.0–13.0). Patients who developed SAP had a significantly smaller percentage decrease in BUN from admission to 24 to 48 hours (*P* = 0.002), SAP median (IQR) decrease of 21.5% (12.5–38.5) versus mild AP group decrease of 35.7% (22.2–52.9). This resulted in BUN values remaining significantly higher (*P* < 0.001) after 24 hours of fluid resuscitation for SAP median 12.5 (8.0–19.0) compared to the mild AP group median 7.0 (5.0–10.0) (Fig. 3). In our validation cohort, a BUN of >20 mg/dL resulted in a 98% specificity of developing SAP, with a PPV of 83% and an NPV of 75% (Supplemental Table 3A, Supplemental Digital Content, http://links.lww.com/MPG/B859), consistent with the previously reported adult findings (6,7). When we examined the BUN change data, we applied this same value to determine whether this would be useful as a cutoff value (Supplemental Table 2, Supplemental Table 3B, Supplemental Digital Content, http://links.lww.com/MPG/B859). In all cohorts and at all time points, applying the cutoff of >20 mg/dL as suggested by the adult literature resulted in a specificity of 98%. Elevated BUN levels within 24 to 48 hours were predictive of developing SAP (AUROC: 0.76, 95% CI: 0.66–0.85) (Supplemental Fig. 1, Supplemental Digital Content, http://links.lww.com/MPG/B859).

## DISCUSSION

Utilizing the 2017 severity classification guidelines, Vitale et al (5) built a model that prospectively predicts the development of SAP based on BUN at admission. We were able to validate this model utilizing a different cohort of patients from 2 separate sites and found that elevated BUN levels on admission were associated with increased severity of disease. Our initial analysis suggested that the addition of albumin optimized the model. In addition, we found that the increases in BUN and decreases in albumin values predicted increasing severity of the disease, from mild to moderately severe AP to SAP.

This model is easy to apply to patient care, involves commonly obtained laboratory samples that can be (and often are) acquired at the time of presentation, and is clinically useful early in presentation (5). These are all characteristics that are generally considered requirements for the development of a strong predictive model (10–12). Optimal validation of the model generally requires an external sample from a similar population to replicate the previously generated results, which we were able to identify at the 2 additional clinical sites, and to generate a discriminating model with a concordance statistic performance that is represented by the area under the ROC curve (10,13,14). An area under the ROC curve of between 0.70 and 0.80 is considered to represent adequate discrimination, whereas >0.80 is considered excellent (10,14). Our BUN only model had an area under the ROC curve of 0.73, very similar to the previously reported results of 0.75, thereby confirming the robustness in replicating the previous values (5). Our model incorporating serum albumin had better prediction, with an area under the curve of 0.83.

Several adult studies have linked elevated BUN levels to increased severity in AP, whether as part of the bedside index for severity of AP scoring or as a stand-alone predictor of severity (6,7,15–21). The proposed mechanism is that the elevated BUN reflects intravascular volume depletion as opposed to renal involvement, given the lack of observed change in the creatinine between the 2 groups (6,17,21). There is also adult data to suggest that decreased albumin on admission is an independent predictor of disease severity in AP as a marker of persistent organ failure, both at admission and then again at 48 hours after admission, likely reflecting the underlying inflammatory state, although the precise mechanism remains unclear (22).

Persistent BUN elevation in our population was also predictive of severity, and BUN elevation at 24–48 hours was an independent predictor of severity in this population. This is a novel finding not previously reported in the pediatric literature. Although it showed similar trends from the adult literature, previous studies did not address the management effects on the BUN change (6,7). We have shown that persistent elevation of BUN was a predictor of SAP independent from rates and types of fluids the patient had received.

Multiple attempts have been made to develop prognostic models for severity in pediatric AP. DeBanto et al (23) developed the Pediatric Acute Pancreatitis Score, which was modeled after adult scoring metrics and involves demographic and biochemical data at admission and then again at 48 hours to calculate the score. In 2013, it was reported that a significantly elevated lipase (≥7× upper limit of normal [ULN]) at time of admission was predictive of severity, but validation of the model had varying sensitivities and specificities (24). Thus, previous attempts to develop a predictive tool in the pediatric population either lacked a common definition of severity or required up to 48 hours from admission to calculate the predictive score, or had varying specificity and sensitivities when replicated in validation studies (23–27). To our knowledge, the first model to use the severity classification consensus published in 2017 and laboratory values on presentation is the BUN model in the derivation cohort and we have now replicated that model successfully (5).

Because of the increased specificity of the predictive model as the BUN level increases, frontline providers should be aware that a higher initial BUN on admission or after initial fluid resuscitation is associated with a higher probability that the patient would develop SAP. In addition, the NPV and PPV can be used to help promote clinical decision making. In all of the models presented, the higher the BUN, the higher the specificity and PPV. The NPV is much higher than the PPV at a lower BUN due to the nature of the severity distribution. When trying to use our model to predict severity, we chose cutoff values that optimize both sensitivity and specificity and PPV and NPV. For instance, in our validation cohort, the model performs optimally at a BUN of 13 mg/dL (sensitivity 68%, specificity 73%). By using a cutoff of 13, we may, however, include some patients who may not develop severe disease. Thus, to interpret BUN as a predictor at admission in our population it may be helpful to think of it in groupings: <13 mg/dL they will most likely have mild AP, 13 to 20 mg/dL there’s a high chance that they will develop SAP so intervention should be considered, and at >20 mg/dL they will almost surely develop SAP so intervention is warranted. This information will allow providers to use commonly performed laboratory tests to augment their clinical decision-making to determine which patients may require an escalation of care or transfer to a specialized facility.

Although our study was successful in validating the utility of BUN as a prognostic indicator of severity in pediatric AP, it is not without limitations. The validation component of this study is limited in size, with only 73 patients across the 2 sites that met the criteria for first episode of AP. We do not have data regarding how long before presentation the patients may have been experiencing symptoms, which may have affected the initial laboratory values for some patients, but this was a “real-world” application of this model. All patients were managed per their respective hospitals’ protocols which may have some institutional variation, are nevertheless similar regarding fluid choices and nutrition. Previous authors have commented on the benefits of avoiding estimation of treatment effects when building prognostic risk prediction models (14).

In conclusion, this study validates a previously generated model, showing that initial BUN is a significant predictor of SAP in the pediatric population. It further optimizes prediction by adding albumin as a significant variable at admission. It also gives another time point for evaluation, allowing clinicians to check BUN at 24 to 48 hours to monitor progression of the disease. Timely identification of high-risk patients will allow referrals to a center that has access to pediatric gastroenterologists with affiliations to a pancreatic center of excellence or a higher level pediatric intensive care unit. Future efforts should focus on combining the predictors of AP into a clinical tool that providers can use to identify the patients at highest risk of progression to severe disease at the time of presentation.

## Supplementary Material

Supplementary Tables and Figures

## Figures and Tables

**TABLE 1.** Baseline demographic, clinical, and biochemical characteristics of validation cohort patients with acute pancreatitis

	SAP (n = 22)	Mild AP (n = 51)	*P*
Age, y	8.5 (7.3–14.4) n = 22	13.3 (8.7–15.4) n = 51	0.17
Sex (female)	12 (55%)	35 (69%)	0.25
BMI percentile	71.6 (58.1–82.1) n = 22	77.3 (45.8–93.9) n = 46	0.53
<5%	4/22 (18%)	2/46 (4%)	
5%–85%	13/22 (59%)	28/46 (61%)	
>85%	5/22 (23%)	16/46 (35%)	
Race			0.51
White/Caucasian	14 (64%)	26 (51%)	
Black/African	6 (27%)	15 (29%)	
American			
Other	2 (9%)	10 (20%)	
LOS, h	147.5 (70.0–348.0) n = 22	94.0 (63.0–154.0) n = 50	0.0497
Lipase × ULN	23.8 (5.6–45.9) n = 21	12.3 (6.9–42.9) n = 48	0.66
Amylase ×ULN	4.1 (2.4–10.5) n = 19	2.2 (1.4–7.1) n = 40	0.15
Albumin, g/dL	3.3 (3.1–4.2) n = 21	4.0 (3.6–4.6) n = 47	0.005
Creatinine, mg/dL	0.5 (0.4–0.7) n = 22	0.5 (0.4–0.7) n = 51	0.94
Calcium, mg/dL	9.2 (8.8–9.5) n = 22	9.3 (8.9–9.7) n = 51	0.29
WBC, 10^3^/μL	13.1 (5.8–17.3) n = 19	9.8 (6.5–13.8) n = 39	0.38
Hematocrit, %	39.4 (33.2–41.9) n = 19	37.3 (34.4–39.8) n 39	0.53
Hemoglobin, g/dL	13.7 (11.0–14.5) n = 19	12.9 (11.7–14.1) n = 39	0.89
BUN, mg/dL	14.5 (11.0–19.0) n = 22	11.0 (8.0–13.0) n = 51	0.002

Data presented as median (25th–75th percentile) or n (%).

AP = acute pancreatitis; BUN = blood urea nitrogen; LOS = length of stay; SAP = severe acute pancreatitis.

## What Is Known

- Severe acute pancreatitis is associated with persistent organ dysfunction and may require interventions not available in a community setting.
- In a single-center study, blood urea nitrogen on admission has been shown as a significant predictor of disease severity.

## What Is New

- In a multicenter study, the role of blood urea nitrogen obtained on admission in predicting progression to severe acute pancreatitis has been validated.
- This model was further optimized with the inclusion of albumin.
- Persistent elevation of blood urea nitrogen after 24 to 48 hours was further associated with progression to severe acute pancreatitis.

## References

[1] Sellers ZM, MacIsaac D, Yu H, Nationwide trends in acute and chronic pancreatitis among privately insured children and non-elderly adults in the United States, 2007–2014. Gastroenterology 2018;155:469.e1–478.e1. 29660323 10.1053/j.gastro.2018.04.013 PMC6067969

[2] Srinath AI, Lowe ME. Pediatric pancreatitis. Pediatr Rev 2013;34:79–90. 23378615 10.1542/pir.34-2-79

[3] Abu-El-Haija M, Kumar S, Quiros JA, Management of acute pancreatitis in the pediatric population: a clinical report from the North American Society for Pediatric Gastroenterology, Hepatology and Nutrition Pancreas Committee. J Pediatr Gastroenterol Nutr 2018;66:159–76. 29280782 10.1097/MPG.0000000000001715 PMC5755713

[4] Abu-El-Haija M, Kumar S, Szabo F, Classification of acute pancreatitis in the pediatric population: clinical report from the NASPGHAN Pancreas Committee. J Pediatr Gastroenterol Nutr 2017;64:984–90. 28333771 10.1097/MPG.0000000000001583

[5] Vitale DS, Hornung L, Lin TK, Blood urea nitrogen elevation is a marker for pediatric severe acute pancreatitis. Pancreas 2019;48:363–6. 30768572 10.1097/MPA.0000000000001265 PMC8579319

[6] Wu BU, Johannes RS, Sun X, Early changes in blood urea nitrogen predict mortality in acute pancreatitis. Gastroenterology 2009;137:129–35. 19344722 10.1053/j.gastro.2009.03.056

[7] Koutroumpakis E, Wu BU, Bakker OJ, Admission hematocrit and rise in blood urea nitrogen at 24 h outperform other laboratory markers in predicting persistent organ failure and pancreatic necrosis in acute pancreatitis: a post hoc analysis of three large prospective databases. Am J Gastroenterol 2015;110:1707–16. 26553208 10.1038/ajg.2015.370

[8] Morinville VD, Husain SZ, Bai H, Definitions of pediatric pancreatitis and survey of present clinical practices. J Pediatr Gastroenterol Nutr 2012;55:261–5. 22357117 10.1097/MPG.0b013e31824f1516 PMC3626452

[9] Hornung RW, Reed LD. Estimation of average concentration in the presence of nondetectable values. Appl Occup Environ Hyg 1990;5:46–51.

[10] Lee YH, Bang H, Kim DJ. How to establish clinical prediction models. Endocrinol Metab (Seoul) 2016;31:38–44. 26996421 10.3803/EnM.2016.31.1.38 PMC4803559

[11] Moons KG, Altman DG, Vergouwe Y, Prognosis and prognostic research: application and impact of prognostic models in clinical practice. BMJ 2009;338:b606. 19502216 10.1136/bmj.b606

[12] Royston P, Moons KG, Altman DG, Prognosis and prognostic research: developing a prognostic model. BMJ 2009;338:b604. 19336487 10.1136/bmj.b604

[13] Altman DG, Vergouwe Y, Royston P, Prognosis and prognostic research: validating a prognostic model. BMJ 2009;338:b605. 19477892 10.1136/bmj.b605

[14] Steyerberg EW, Vergouwe Y. Towards better clinical prediction models: seven steps for development and an ABCD for validation. Eur Heart J 2014;35:1925–31. 24898551 10.1093/eurheartj/ehu207 PMC4155437

[15] Faisst M, Wellner UF, Utzolino S, Elevated blood urea nitrogen is an independent risk factor of prolonged intensive care unit stay due to acute necrotizing pancreatitis. J Crit Care 2010;25:105–11. 19427764 10.1016/j.jcrc.2009.02.002

[16] Kaya E, Dervisoglu A, Polat C. Evaluation of diagnostic findings and scoring systems in outcome prediction in acute pancreatitis. World J Gastroenterol 2007;13:3090–4. 17589925 10.3748/wjg.v13.i22.3090 PMC4172616

[17] Lin S, Hong W, Basharat Z, Blood urea nitrogen as a predictor of severe acute pancreatitis based on the revised Atlanta criteria: timing of measurement and cutoff points. Can J Gastroenterol Hepatol 2017;2017:9592831. 28487848 10.1155/2017/9592831 PMC5406719

[18] Liu G, Tao J, Zhu Z, The early prognostic value of inflammatory markers in patients with acute pancreatitis. Clin Res Hepatol Gastroenterol 2019;43:330–72019. 30545732 10.1016/j.clinre.2018.11.002

[19] Valverde-Lopez F, Matas-Cobos AM, Alegria-Motte C, BISAP, RANSON, lactate and others biomarkers in prediction of severe acute pancreatitis in a European cohort. J Gastroenterol Hepatol 2017;32:1649–56. 28207167 10.1111/jgh.13763

[20] Vasudevan S, Goswami P, Sonika U, Comparison of various scoring systems and biochemical markers in predicting the outcome in acute pancreatitis. Pancreas 2018;47:65–71. 29215536 10.1097/MPA.0000000000000957

[21] Wu BU, Bakker OJ, Papachristou GI, Blood urea nitrogen in the early assessment of acute pancreatitis: an international validation study. Arch Intern Med 2011;171:669–76. 21482842 10.1001/archinternmed.2011.126

[22] Li S, Zhang Y, Li M, Serum albumin, a good indicator of persistent organ failure in acute pancreatitis. BMC Gastroenterol 2017;17:59. 28446147 10.1186/s12876-017-0615-8 PMC5406910

[23] DeBanto JR, Goday PS, Pedroso MR, Acute pancreatitis in children. Am J Gastroenterol 2002;97:1726–31. 12135026 10.1111/j.1572-0241.2002.05833.x

[24] Coffey MJ, Nightingale S, Ooi CY. Serum lipase as an early predictor of severity in pediatric acute pancreatitis. J Pediatr Gastroenterol Nutr 2013;56:602–8. 23403441 10.1097/MPG.0b013e31828b36d8

[25] Izquierdo YE, Fonseca EV, Moreno LA, Multivariate model for the prediction of severity of acute pancreatitis in children. J Pediatr Gastroenterol Nutr 2018;66:949–52. 29601435 10.1097/MPG.0000000000001983

[26] Lautz TB, Chin AC, Radhakrishnan J. Acute pancreatitis in children: spectrum of disease and predictors of severity. J Pediatr Surg 2011;46:1144–9. 21683213 10.1016/j.jpedsurg.2011.03.044

[27] Szabo FK, Hornung L, Oparaji JA, A prognostic tool to predict severe acute pancreatitis in pediatrics. Pancreatology 2016;16:358–64. 27051062 10.1016/j.pan.2016.03.002

