# Supplementary material for: Who’s at Risk? A Prognostic Model for Severity Prediction in Pediatric Acute Pancreatitis
Source: J Pediatr Gastroenterol Nutr. Author manuscript; Available in PMC 2021 Apr 5. (PMC8020899; doi:10.1097/MPG.0000000000002807)

Supplemental Table 1: Complete list of the admission biochemical characteristics of validation cohort patients with AP

	SAP (n=22)	Mild AP (n=51)	*p*-value
**Lipase x ULN**	23.8 (5.6-45.9) *n=21*	12.3 (6.9-42.9) *n=48*	0.66
**Amylase x ULN**	4.1 (2.4-10.5) *n=19*	2.2 (1.4-7.1) *n=40*	0.15
**Albumin, g/dL**	3.3 (3.1-4.2) *n=21*	4.0 (3.6-4.6) *n=47*	**0.005**
**Anion Gap, mmol/L**	12.0 (8.0-16.0) *n=21*	12.0 (10.0-15.0) *n=51*	0.74
**WBC, 10^3/microL**	13.1 (5.8-17.3) *n=19*	9.8 (6.5-13.8) *n=39*	0.38
**Creatinine, mg/dL**	0.5 (0.4-0.7) *n=22*	0.5 (0.4-0.7) *n=51*	0.94
**Calcium, mg/dL**	9.2 (8.8-9.5) *n=22*	9.3 (8.9-9.7) *n=51*	0.29
**AST, Unit/L**	32.0 (24.0-63.0) *n=21*	38.0 (22.0-80.0) *n=47*	0.69
**ALT, Unit/L**	29.0 (21.0-57.0) *n=21*	31.0 (24.0-135.0) *n=47*	0.39
**Hematocrit, %**	39.4 (33.2-41.9) *n=19*	37.3 (34.4-39.8) *n=39*	0.53
**Hemoglobin, g/dL**	13.7 (11.0-14.5) *n=19*	12.9 (11.7-14.1) *n=39*	0.89
**BUN, mg/dL**	14.5 (11.0-19.0) *n=22*	11.0 (8.0-13.0) *n=51*	**0.002**
**Alk Phosphorus, Unit/L**	163.0 (137.0-207.0) *n=21*	138.5 (91.0-225.0) *n=46*	0.54
**Glucose, mg/dL**	97.5 (80.0-138.0) *n=22*	99.0 (89.0-118.0) *n=51*	0.75
**Triglyceride, mg/dL**	49.5 (35.0-69.0) *n=10*	68.0 (50.0-115.0) *n=21*	0.06
**Sodium, mmol/L**	138.5 (136.0-140.0) *n=22*	140.0 (138.0-142.0) *n=51*	0.11
**Chloride, mmol/L**	103.5 (101.0-106.0) *n=22*	104.0 (101.0-106.0) *n=51*	0.91
**Total Bilirubin, mg/dL**	0.5 (0.4-0.9) *n=21*	0.6 (0.4-1.1) *n=47*	0.50
**CRP, mg/dL**	1.3 (0.5-6.6) *n=11*	1.1 (0.6-3.0) *n=29*	0.83
**Potassium, mmol/L**	4.1 (3.9-4.6) *n=21*	4.1 (3.7-4.4) *n=51*	0.64
**Total protein, g/dL**	6.6 (5.9-7.6) *n=21*	7.1 (6.5-7.5) *n=47*	0.20
**Platelet count, 10^3/microL**	288.0 (154.0-393.0) *n=19*	261.0 (219.0-326.0) *n=39*	0.67
**CO2, mmol/L**	24.5 (18.0-27.0) *n=22*	24.0 (22.0-25.0) *n=51*	0.93

Data presented as median (25th-75th percentile) or n (%)

Supplemental Table 2: BUN Change admission to 24-48 hours, patient characteristics

	All<br>N=176	Mild AP<br>N=137	SAP*<br>N=39	P-value†
**Age at admission (years)**	13.5 (8.7, 15.6)	13.5 (9.3, 15.6)	12.3 (7.7, 15.5)	0.29
**Sex (female)**	96 (55%)	77 (56%)	19 (49%)	0.41
**Fluid type**				0.37
**Isotonic**	58/157 (37%)	47/121 (39%)	11/36 (31%)	
**Hypotonic**	88/157 (56%)	67/121 (55%)	21/36 (58%)	
**TPN/Other**	11/157 (7%)	7/121 (6%)	4/36 (11%)	
**Fluid rate**				0.73
**< 1.5x maintenance**	76/160 (47%)	58/122 (48%)	18/38 (47%)	
**1.5 - <2x maintenance**	70/160 (44%)	52/122 (43%)	18/38 (47%)	
**≥ 2x maintenance**	14/160 (9%)	12/122 (10%)	2/38 (5%)	
**Feeds on admission**				-
**NPO**	134/165 (81%)	101/128 (79%)	33/37 (89%)	
**Clear liquid diet**	17/165 (10%)	16/128 (13%)	1/37 (3%)	
**PO general diet**	10/165 (6%)	7/128 (5%)	3/37 (8%)	
**PO low fat diet**	3/165 (2%)	3/128 (2%)	0/37 (0%)	
**Feeds through existing enteral tube**	1/165 (1%)	1/128 (1%)	0/37 (0%)	
**IV bolus within 6 hours**	103/162 (64%)	75/125 (60%)	28/37 (76%)	0.08
**BUN admission (mg/dL)**	11.0 (8.0, 15.0)	10.0 (8.0-13.0)	15.5 (11.0-22.5)	<0.0001
**BUN 24-48hrs (mg/dL)**	8.0 (6.0, 12.0)	7.0 (5.0-10.0)	12.5 (8.0-19.0)	<0.0001
**BUN change (mg/dL)**	-3.0 (-6.0, -1.0)	-3.0 (-5.0, -1.0)	-3.5 (-7.0, -1.0)	0.32
**BUN no change admission-24hrs**	15/143 (10%)	13/109 (12%)	2/34 (6%)	0.52
**BUN percent change from admission (includes all)**	-23.5 (-44.4, -9.1)<br>*n=143*	-27.3 (-50.0, -9.1)<br>*n=109*	-19.1 (-32.4, -9.4)<br>*n=34*	0.22
**BUN percent rise from admission (for those increased)**	25.0 (10.0, 40.0)<br>*n=15*	25.0 (16.7, 40.0)<br>*n=13*	19.8 (6.3, 33.3)<br>*n=2*	0.60
**BUN percent decrease from admission (for those decreased)**	-33.3 (-50.0, -20.0)<br>*n=113*	-35.7 (-52.9, -22.2)<br>*n=83*	-21.5 (-38.5, -12.5)<br>*n=30*	0.002
**BUN elevated admission (>20 mg/dL)**	15/162 (9%)	3/126 (2%)	12/36 (33%)	<0.0001
**BUN elevated 24hrs (>20 mg/dL)**	10/145 (7%)	2/111 (2%)	8/34 (24%)	0.0001
**BUN elevated both at admission & at 24 hours (>20 mg/dL)**	10/143 (7%)	2/109 (2%)	8/34 (24%)	0.0002

Data presented as median (25th-75th percentile) or n (%).

*SAP (moderately severe and severe AP)

†P-value is from testing differences between the Mild AP and SAP groups

Supplemental Table 3: BUN Values and Resultant Statistics

A. Derived from the CHKD and CNH Data (n= 73)

BUN Value (mg/dL)	Sensitivity	Specificity	PPV	NPV
**10**	77%	33%	33%	77%
**11**	77%	49%	40%	83%
**12**	73%	59%	43%	83%
**13**	68%	73%	52%	84%
**14**	59%	76%	52%	81%
**15**	50%	82%	55%	79%
**16**	45%	90%	67%	79%
**17**	41%	90%	64%	78%
**18**	36%	92%	67%	77%
**19**	27%	94%	67%	75%
**21**	23%	98%	83%	75%
**23**	18%	98%	80%	74%
**24**	14%	98%	75%	72%
**37**	14%	100%	100%	73%

B. Derived from the BUN Change Cohort (CHKD & CCHMC) data (n=176)

	Admission				24-48 hours			
**BUN Value (mg/dL)**	**Sensitivity**	**Specificity**	**PPV**	**NPV**	**Sensitivity**	**Specificity**	**PPV**	**NPV**
**10**	83%	38%	28%	89%	59%	71%	38%	85%
**11**	78%	52%	31%	89%	59%	81%	49%	87%
**12**	72%	60%	34%	88%	56%	84%	51%	86%
**13**	69%	72%	42%	89%	50%	88%	57%	85%
**14**	61%	79%	45%	88%	44%	94%	68%	85%
**15**	56%	83%	48%	87%	41%	95%	70%	84%
**16**	50%	88%	55%	86%	41%	95%	74%	84%
**17**	47%	88%	53%	85%	35%	95%	71%	83%
**18**	44%	91%	59%	85%	32%	95%	69%	82%
**19**	42%	91%	59%	85%	26%	96%	69%	81%
**20**	39%	94%	67%	84%	24%	97%	73%	81%
**21**	33%	98%	80%	84%	24%	98%	80%	81%
**22**	28%	98%	77%	83%	21%	98%	78%	80%
**25**	19%	98%	70%	81%	18%	98%	75%	80%
**32**	17%	99%	86%	81%	NA	NA	NA	NA

Supplemental Figure 1.

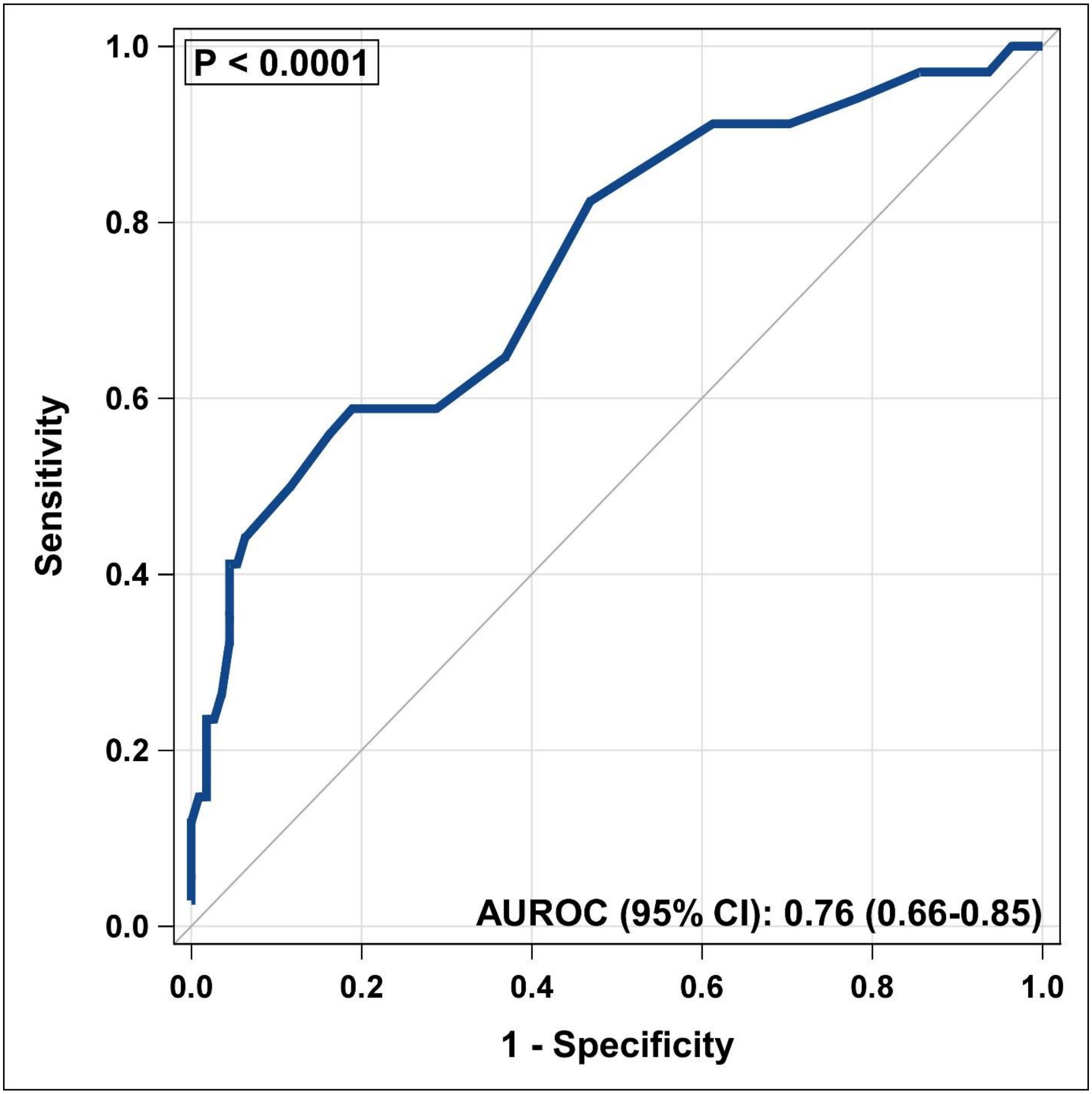

Supplement: Supplementary Tables and Figures [file NIHMS1682481-supplement-Supplementary_Tables_and_Figures.pdf]
